# Glucose and lipid lowering effects of *Enhydra fluctuans* extract in cadmium treated normal and type-2 diabetic model rats

**DOI:** 10.1186/s12906-019-2667-5

**Published:** 2019-10-22

**Authors:** Mohammad Nazmul Hasan, Farah Sabrin, Begum Rokeya, Md Shahinul Haque Khan, Mahtab Uddin Ahmed, Abel Matondo, Md Morsaline Billah, Salima Akter

**Affiliations:** 1 Department of Biotechnology and Genetic Engineering, Mawlana Bhashani Science and Technology University, Santosh, Tangail, 1902 Bangladesh; 2 Department of Pharmacology, Bangladesh University of Health Sciences, Dhaka, 1216 Bangladesh; 3 Department of Chemistry, Bangladesh University of Health Sciences, Dhaka, 1216 Bangladesh; 4 Department of Pathology, Bangladesh University of Health Sciences, Dhaka, 1216 Bangladesh; 5 Department of Health and Social Care, St. Patrick’s College, London, UK; 6 Biotechnology and Genetic Engineering Discipline, Khulna University, Khulna, 9208 Bangladesh; 7 Department of Medical Biotechnology, Bangladesh University of Health Sciences, Dhaka, 1216 Bangladesh

**Keywords:** Hyperglycemia, Dyslipidemia, Type 2 diabetes, Cadmium toxicity, *Enhydra fluctuans*

## Abstract

**Background:**

Recent epidemiological and experimental studies suggest that cadmium and diabetes-related hyperglycemia may act synergistically to worsen metabolic regulation. The present study aims to evaluate the potential effects of *Enhydra fluctuans* extract in diabetes and dyslipidemia in cadmium (CdCl_2_) induced- normal and type 2 diabetic model rats.

**Method:**

Forty-eight Long-Evans rats were divided equally into the following six groups: Normal Control (N-C), Normal treated with CdCl_2_ (N-Cd), Normal treated with plant extract (N-P), Normal treated with both plant extract and CdCl_2_ (N-PCd), Diabetic treated with plant extract (DM-P) and Diabetic treated with both plant extract and CdCl2 (DM-PCd). Blood glucose and other biochemical parameters were estimated by the enzymatic colorimetric method. Histological analysis of liver and heart was done by the hematoxylin-eosin (H & E) method.

**Results:**

Twenty-one days treatment of *E. fluctuans* extracts at a dose of 200 mg/kg significantly reduced blood glucose level in N-PCd and DM-PCd (*p* < 0.05), and DM-P (*p* < 0.01) group. The plant extract had no direct effects on total blood lipids but, it had beneficial effects on TG/HDL-C ratio in N-P and DM-PCd groups (p < 0.05). Cd induction significantly reduced body weight [(N-Cd, N-PCd, DM-PCd) (p < 0.01)], and induced liver [N-Cd (p < 0.05), N-PCd, *p* < 0.001] and renal impairment [N-Cd (p < 0.05)]. In bi-variate association, a significant positive correlation between serum glucose and SGPT (p < 0.05) as well as SGPT and TG/HDL ratio (*p* = 0.019) was found in DM-P and in the merged group. The histology of liver and heart showed severe damages including inflammation, nuclear pyknosis, loss of myocardial fibers, necrosis and fibrosis in the Cd treated groups compared to plant treated groups.

**Conclusion:**

*E. fluctuans* seems to have potent antihyperglycemic effects in diabetes and Cd toxicity along with partial antidyslipidemic properties in euglycemic and diabetic rats. Our study suggests a novel oral antihyperglycemic agent in the present environmental context.

## Background

Diabetes mellitus (DM) is a chronic metabolic disorder characterized by elevated levels of glucose in the blood. It is one of the rapid rising health challenges in middle- and low-income countries. According to the WHO, the worldwide prevalence of DM in adults was estimated to 4.7% in 1980, and 8.5% in 2014 [1, 2]. In 2016, 1.6 million diabetes-associated deaths were recorded, and currently 422 million people live with diabetes related complications characterized by microvascular (viz. retinopathy, neuropathy and nephropathy) or macrovascular (viz. myocardial infarction, heart failure and stroke) diseases resulting from chronic exposure to high glucose and altered blood lipids [3, 4]. Predominant factors like sedentary high-stress lifestyle and poor diet including high glycemic and trans-fat with low-fiber and -phytonutrients contribute to the epidemic of diabetes [5]. Moreover, the increasing burden of environmental toxins, including persistent organic pollutants and heavy metals, can no longer be ignored as a key etiologic factors of this condition [6, 7].

Among the heavy metals, cadmium is the most abundant environmental pollutant, which comes from industrial wastes, cigarette smoking, electroplating, plastic products, pigments, phosphate fertilizer, battery manufactures and pesticides [8]. It has a long half-life that disperses in the soil and persists for decades. Therefore, Cd can easily enter into the food chain or efficiently transfer to plants [9]. This toxic heavy metal has many adverse health effects [10] and studies have suggested that Cd induced different types of cancers [11, 12], liver- and renal dysfunction [13], diabetes and cardiovascular diseases [14]. In diabetes, it exacerbates beta cell dysfunction and studies showed varied results [15–17]. In the liver, sub-chronic Cd exposure has been shown to increase the activity of enzymes responsible for gluconeogenesis and altered lipid metabolism [18]. Also in the kidney it exacerbates renal failure, and synergistic action has been demonstrated between Cd induced renal dysfunction and diabetes [19, 20].

Currently, the available therapy for diabetes comprises insulin and various oral antidiabetic agents namely thiazolidinediones, sulfonylureas and α-glucosidase inhibitors. However, most oral antidiabetic agents have their own limitations and adverse side effects [21–23]. To overcome these limitations, a great attention has been focused on a number of potential molecular targets. Recently, plant-derived extracts or compounds were evaluated to have antidiabetic targets such as α-glucosidase, α-amylase, DPP-4, PPAR-γ, PTP1B and GLUT4 [24]. Hence, antidiabetic drug discovery has altered its focus to natural medicinal plant sources due to nominal side effects [25, 26].

In this study, we evaluated *Enhydra fluctuans* (Family: Asteraceae), a trailing marsh herb, commonly known as ‘Helencha’ in Bangladesh. The plant contains various phytochemical compounds such as flavonoids, alkaloids, saponins, tannins, phenols and carbohydrates [27, 28]. The hydrophytes are capable of removing the majority of pollutants particularly heavy metals from wastewater by sorption, sedimentation and assimilation processes [29]. In ethnopharmacology, *E. fluctuans* has been well studied as a CNS depressant, analgesic, anticancer, hepatoprotective and anthelmintic agent. [27, 30, 31]. However, there is no experimental evidence regarding the effects of *E. fluctuans* plants in hyperglycemia and hyperdyslipidemia. Some traditional medicine practitioner’s use *E. fluctuans* as an antidiabetic plant in tribal populations [32]. Therefore, it was hypothesized that *E. fluctuans* may reduce blood sugar and lipids levels, and has the ability to retain its effect in heavy metal (Cd) toxicity. In this context, we investigated the antidiabetic and antidyslipidemic effects of *E. fluctuans* extract in Cd treated normal and type 2 diabetic rat models, and explored whether the plant could protect Cd and diabetes related complications.

## Methods

### Chemicals and reagents

Streptozotocin was obtained from Sigma Aldrich (St. Louis, MO, USA). Cadmium chloride (CdCl_2_) was purchased from Qualikems Fine Chemicals Ltd. (New Delhi, India). Dimethyl sulfoxide (DMSO) was obtained from Sigma. All other chemicals used were purchased from Merck Chemicals (Germany).

### Plant collection and processing

The whole plant (30 kg of (E. fluctuans)) was collected from fields and canal sides of Maynamati, Cumilla, in the month of November 2016. The National Herbarium Institute (BNHI), Ministry of Environment and Forests Dhaka, Bangladesh identified the plant. The accession number is DACB 46953. The plant materials were sundried and 10 kg of dried plant was obtained. Then the dried materials were grinded to powder and sieved with 40 mesh size. The two kilograms of fine powder obtained, were used for extraction with 4 l of 20% aqueous alcohol by keeping overnight, the extraction process was repeated thrice (4 l × 3). The mixture was filtered by Whatman filter paper (No. 1441090) and a total 9.0 l of filtrate was obtained. The filtrate was then evaporated using a vacuum rotary evaporator not exceeding 40 °C. The semi-dried extract was further dried in a freeze drier. The dried material was obtained and stored in an airtight container at − 20 °C for further analysis.

### Experimental animal

Forty eight adult long evans rats used in the study, 32 were normal and 16 were type 2 diabetic rats weighing 200–250 g. The animals were bred at Bangladesh University of Health Sciences (BUHS) animal house, Dhaka, Bangladesh. Animals were housed in 20.0 cm × 15.0 cm × 7.0 cm (Length x Width x Height respectively) cages. Then allowed to acclimatize for seven days in the environment maintained at a constant room temperature of 22 ± 5 °C with humidity of 40–70% and the natural 12 h day-night cycle [33]. The animal environment, housing, and management were strictly followed by standard guideline [34]. The rats were fed on a standard laboratory pellet diet and purified water supplied ad libitum. The influence of circadian rhythm was avoided by performing the experiments in the morning. The ethical approval for the study was taken from the Ethical Committee of BUHS, Bangladesh (Reg. no. BUHS/BIO/EA/18/11).

### Experimental model of diabetes

Type 2 diabetes was induced by a single intra peritoneal injection of Streptozotocin (STZ) in citrate buffer (10 mL) at a dose of 90 mg/kg body weight (bw) to the rat pups (48 h old, average weight 7 g) [35]. The three months old rats were primary assessed following standard guideline of oral glucose tolerance test (OGTT). Streptozotocin induced diabetic model is not the actual type 2 DM model [36]; therefore, our study also included 40% rats that developed diabetes naturally. The final experimental rats were considered based on fasting blood glucose level > 7.0 mM of STZ treatment and > 8.0 mM of naturally DM on day 0. These rats were distributed equally in both DM-P and DM-PCd groups.

### Experimental design

The animals were divided into the following six groups, four of which were normal and two were diabetic models containing eight (*n* = 8) in each group. The experiment was carried out for 21 days study period.

- **N-C group:** Normal rats with water and dimethyl sulphooxide (DMSO) [10 mg/kg bw].
- **N-Cd group:** Normal rats with Cadmium chloride (CdCl_2_) treated group [50 mg/kg bw].
- **N-P group:** Normal rats with plant treated (NP) group. *Enhydra fluctuans* extract (200 mg/kg bw).
- **N-PCd group:** Normal rats with plant (NP) + CdCl_2_ treated group. CdCl_2_ (50 mg/kg bw) + *Enhydra fluctuans* extract (200 mg/kg bw).
- **DM-P group:** Diabetic rats with plant treated group. *Enhydra fluctuans* extract (200 mg/kg bw) was orally administered.
- **DM-PCd group:** Diabetic rats with CdCl_2 +_ plant treated group. CdCl_2_ (50 mg/kg bw) + *Enhydra fluctuans* extract (200 mg/kg bw).

All the treatment regiments were administered orally. Health, food and water intake were monitored daily whilst body weight was determined weekly. After overnight fasting, blood samples (0.5 mL) were collected from tail-vein using mild ether anesthesia (5.6 g/kg) at day 0 and day 21 of treatment period [37]. The fasting blood glucose levels and other biochemical parameters namely total cholesterol (TC), triglycerides (TG), high-density lipoprotein (HDL), alanine amino transferase (ALT) and creatinine levels were estimated using serum samples.

All the six groups of rats were sacrificed on day 21 after an overnight fast, with anesthetic ether and further by cervical dislocation. After sacrificing, liver and heart samples were immediately collected and stored at 10% formalin for histological analysis.

### Biochemical parameters

#### Estimation of glucose

Serum glucose was measured by the glucose oxidase (GOD-PAP) method [38], which uses oxidation of glucose to generate gluconic acid and hydrogen peroxide. Hydrogen peroxide (H_2_O_2_) forms a red violet color with a chromogenic oxygen acceptor, phenol aminophenazone in the presence of peroxidase (POD). The color intensity is proportional to glucose concentration in the sample.

#### Estimation of cholesterol

Serum cholesterol level of the rats was determined after enzymatic hydrolysis and oxidation. The indicator quinoneimine was generated from hydrogen peroxide and 4-aminoantipyrine in the presence of phenol and peroxidase [39].

#### Estimation of serum triglyceride (TG)

The serum triglyceride level was estimated by GPO-PAP method using the enzyme glycerol-3-phosphate oxidase by Automated Analyzer [40].

#### Determination of HDL-cholesterol

Serum HDL-Cholesterol was determined using the method described previously [39].

#### Calculation of LDL- cholesterol

Low density lipoprotein (LDL)-cholesterol was calculated according to the previously described formula [41].

#### Atherogenic index

Atherogenic index was calculated using the formula, total TG/HDL-C [42].

#### Estimation of alanine aminotransferase

It is an adaptation of alanine aminotransferase procedure of the IFCC as described previously [43]. This procedure is modified by adding of pyridoxal-5-phosphate (P5P) as an activator to replace phosphate buffer with tris (hydroxymethyl) aminomethane.

#### Estimation of creatinine

The serum creatinine level was measured using a modification of kinetic Jaffe reaction [44].

All serum biochemical parameters were estimated by enzymatic colorimetric method using Dimension® clinical chemistry system, SIMENS, Germany.

### Histological analysis

For histological studies, liver and heart portions were excised and fixed in 10% formalin for 24 h. After fixing the tissue, it was dehydrated by a graded series of ethyl alcohol (70 to 100%) and cleared by toluene, then embedded in paraffin wax. Sections of paraffin blocks were cut by a rotary microtome (5 μm) Leica 2235 scanner (Germany) and stained with haematoxylin and eosin (H&E) [45]. The samples were examined under a light microscope (Zeiss Axio Scope.A1, Germany) and images were taken at 20X and 40X magnification.

### Statistical analysis

Statistical analysis was performed by Statistical Package for Social Sciences (software for windows version 16 (SPSS Inc., Chicago, Illinois, USA). Data were expressed as mean ± standard deviation or median (range). Comparison within groups was performed using paired t-test while one-way ANOVA followed by Dunnett’s test were used between groups. To estimate the correlation between dependent and independent variables, Pearson’s correlation was perfomed, a *p* < 0.05 was considered statistically significant.

## Results

### Glycemic status of the study subjects

In the six rat models, fasting serum glucose (FSG) levels were comparable within normal models and diabetic models on day 0. After oral administration of respective treatments for 21 days, the corresponding FSG level was significantly reduced in N-PCd, DM-PCd and DM-P groups [mean ± SD, mmol/l; (6.71 ± 0.97, 8.22 ± 0.83 and 7.80 ± 0.44) day 0 vs (6.24 ± 0.92, 6.74 ± 1.45 and 6.70 ± 0.44) day 21 respectively; p < 0.05 and *p* < 0.01] **(**Table 1**)**. So, *E. fluctuans* plant retained potent glucose lowering effects both in hyperglycemia and in Cd toxicity. The comparative analyses among the different groups at baseline and endpoint of the study were also performed. The baseline glucose was significantly different among the study groups confirmed by F test (F = 7.841, *p* < 0.001), however, the significance level disappeared at endpoint of the treatment **(**Table 2**)**.

**Table 1:** Effects of *E. fluctuans* extract on glycemic levels in the study groups

Parameter	N-C (*n* = 8)	N-Cd (*n* = 7)	N-P (*n* = 7)	N-PCd (*n* = 7)	DM-P (*n* = 6)	DMP-Cd (*n* = 5)
Fasting Serum Glucose (mM)						
Day 0	5.59 ± 0.87	6.75 ± 1.19	6.37 ± 0.69	6.71 ± 0.97	7.80 ± 0.44	8.22 ± 0.83
Day 21	6.04 ± 0.91	6.75 ± 0.74	6.31 ± 1.18	6.24 ± 0.92^*^	6.70 ± 0.41 ^**^	6.74 ± 1.45 ^*^

Results are expressed as Mean ± SD. Statistical analysis within groups was done using paired t-test. *SD* Standard deviation, *N-C* Normal Control, *N-Cd* Normal treated with CdCl_2_, *N-P* Normal treated with plant extract; *N-PCd* Normal treated with both plant extract and CdCl_2_, *DM-P* Diabetic treated with plant extract, *DM-PCd* Diabetic treated with both plant extract and CdCl_2_. *, ** and *** indicate statistically significant (*p* < 0.05, *p* < 0.01 and < 0.001) level from a paired t-test

**Table 2:** Comparison of glycemic status before and after *E. fluctuans* treatments

Group	Before Treatment				After Treatment		
	FSG (mM) Mean ± SD	Mean difference ± SE	F ratio (Significance)	*p* value	FSG (mM) Mean ± SD	Mean difference ± SE	F ratio (Significance)
N-C	5.59 ± 0.87		7.841 (*p* < 0.001)		6.04 ± 0.91		0.647 (*p* = 0.666)
N-Cd	6.75 ± 1.19	1.16 ± 0.47		0.072	6.75 ± 0.74	0.71 ± 0.53	
N-P	6.37 ± 0.69	0.79 ± 0.43		0.270	6.31 ± 1.18	0.28 ± 0.49	
N-PCd	6.71 ± 0.98	1.13 ± 0.45		0.067	6.24 ± 0.92	0.21 ± 0.51	
DM-P	7.80 ± 0.44	2.20 ± 0.47		< 0.001^***^	6.70 ± 0.41	0.66 ± 0.53	
DMP-Cd	8.22 ± 0.83	2.63 ± 0.49		< 0.001^***^	6.74 ± 1.45	0.70 ± 0.56	

Results are expressed as Mean ± SD and Mean difference ± SE. Mean differences were calculated in comparison to Control group (N-C). *SD* Standard deviation, *SE* Standard error of mean, *FSG* Fasting serum glucose. *** indicates statistically significant (*p* < 0.001) level from one-way ANOVA and Dunnett’s test

### Effects of *E. fluctuans* on serum cholesterol, triglyceride, HDL-cholesterol and atherogenic index

In the lipidemic status, none of the six groups showed significant difference regarding serum total cholesterol, TG and HDL-C between day 0 and day 21 **(**Table 3). However, the atherogenic index calculated from the ratio of TG to HDL-C showed significant difference from the day 21 to baseline index. Ethanolic extracts significantly decreased serum TG to HDL-C ratio in N-P and DM-PCd groups (*p* < 0.05).

**Table 3:** Effects of *E. fluctuans* on lipid profile and atherogenic index in the study subjects

Para-meters	Days	N-C	N-Cd	N-P	N-PCd	DM-P	DMP-Cd
Chol (mg/dl)	0 day	75 ± 19	72 ± 18	76 ± 18	73 ± 15	75 ± 15	75 ± 10
	21 day	67 ± 8	75 ± 8	68 ± 7	68 ± 15	84 ± 12	73 ± 13
TG (mg/dl)	0 day	58 ± 18	57 ± 15	55 ± 13	62 ± 21	53 ± 5	83 ± 12
	21 day	51 ± 8	50 ± 12	48 ± 18	61 ± 18	56 ± 10	56 ± 12
HDL-C (mg/dl)	0 day	65 ± 15	64 ± 16	59 ± 9	65 ± 17	67 ± 14	58 ± 4
	21 day	65 ± 11	70 ± 7	64 ± 7	63 ± 15	78 ± 10	68 ± 13
TG/HDL-C ratio	0 day	0.75 (0.49–2.38)	0.84(0.62–1.41)	0.89(0.74–1.24)	0.90(0.59–1.92)	0.83(0.66–0.98)	1.57(1.07–1.64)
	21 day	0.74(0.60–1.15)	0.65(0.60–1.12)	0.66(0.48–1.27)*	0.82(0.62–1.89)	0.74(0.47–0.96)	0.81(0.63–1.08)*

Results are expressed as Mean ± SD or Median (range) as appropriate. Statistical analysis within groups was done using paired t-test and WilCoxon rank sum test. SD, Standard deviation; * and ** indicate statistically significant (*p* < 0.05 and *p* < 0.01) levels from a paired t-test

### Effects of *E. fluctuans* extracts and cadmium on body weight, liver and kidney

As for body weight measurement, all cadmium treated groups (N-Cd, N-PCd and DM-PCd) showed a significant reduction in body weight on day 21 (*p* < 0.01). In contrast, no significant changes in body weight were observed in non-Cd-treated (N-C, N-P and DM-P) groups (p = ns). To compare the deleterious effects of Cd and plant on liver and kidney, serum GPT and creatinine levels among the six groups on day 0 and day 21 were depicted in Table 4. Liver function was significantly impaired in N-PCd (*p* < 0.001) and Cd treated groups N-Cd (p < 0.05), but kidney impairment was only observed in N-Cd (p < 0.05) group **(**Table 4**)**.

**Table 4:** Effects of *E. fluctuans* and cadmium on body weight, liver and kidney function

Parameters	Days	N-C	N-Cd	N-P	N-PCd	DM-P	DM-PCd
Weight (g)	0 day	225 ± 14	223 ± 10	226 ± 13	234 ± 14	231 ± 13	220 ± 16
	21 day	217 ± 16	203 ± 10**	221 ± 19	201 ± 25**	221 ± 10	187 ± 24**
SGPT (mg/dl)	0 day	58 ± 19	59 ± 8	53 ± 10	73 ± 29	72 ± 40	62 ± 13
	21 day	75 ± 15	106 ± 39*	69 ± 21	123 ± 37***	87 ± 33	68 ± 13
Creatinine (mg/dl)	0 day	0.51 ± 0.06	0.48 ± 0.10	0.51 ± 0.06	0.56 ± 0.05	0.57 ± 0.08	0.56 ± 0.15
	21 day	0.59 ± 0.08	0.60 ± 0.09*	0.58 ± 0.07	0.63 ± 0.07	0.52 ± 0.04	0.46 ± 0.05

Results are expressed as Mean ± SD. Statistical analysis within groups was done using paired t-test. SD, Standard deviation; *, ** and *** indicate statistically significant (*p* < 0.05, *p* < 0.01 and *p* < 0.001) levels from a paired t-test

### Correlation

Several significant correlations were observed when different groups were analyzed. One of the most important correlation was the association of FSG with serum GPT on day 21 in DM-P (r = 0.920, *p* = 0.009) and the merged group (r = 0.313, *p* = 0.043) **(**Table 5**)**. This association was also found (*p* = 0.05) in N-PCd at baseline that disappeared after 21 days of treatment period. No significant correlation was observed from the remaining groups either on day 0 or day 21 (p = ns). The correlation between SGPT and TG/HDL-C on day 21 was found to be significantly associated with the merged group (*p* < 0.01), NP and DMP (< 0.05) **(**Table 5**)**.

**Table 5:** The association of fasting serum glucose, SGPT and TG/HDL ratio in the study groups

Groups	FSG vs SGPT		SGPT vs TG/HDL ratio	
	Day 0 (r)	Day 21 (r)	Day 0 (r)	Day 21 (r)
Merged group	+ 0.121	+ 0.313^a^	+ 0.056	+ 0.410^b^
N-C	−0.459	+ 0.569	−0.100	−0.291
N-Cd	+ 0.188	+ 0.728	−0.113	−0.675
N-P	−0.464	+ 0.351	+ 0.498	+ 0.836^a^
N-PCd	+ 0.755^a^	+ 0.404	+ 0.277	+ 0.681
DM-P	−0.592	+ 0.920^b^	+ 0.314	+ 0.882^a^
DM-PCd	−0.188	−0.179	+ 0.122	− 0.339

r, Correlation coefficient; ^a^ and ^b^ indicate significance at 0.05 and 0.01 levels (2-tailed)

### Effects of *E. fluctuans* extract on liver and heart histology

**Fig. 1:**
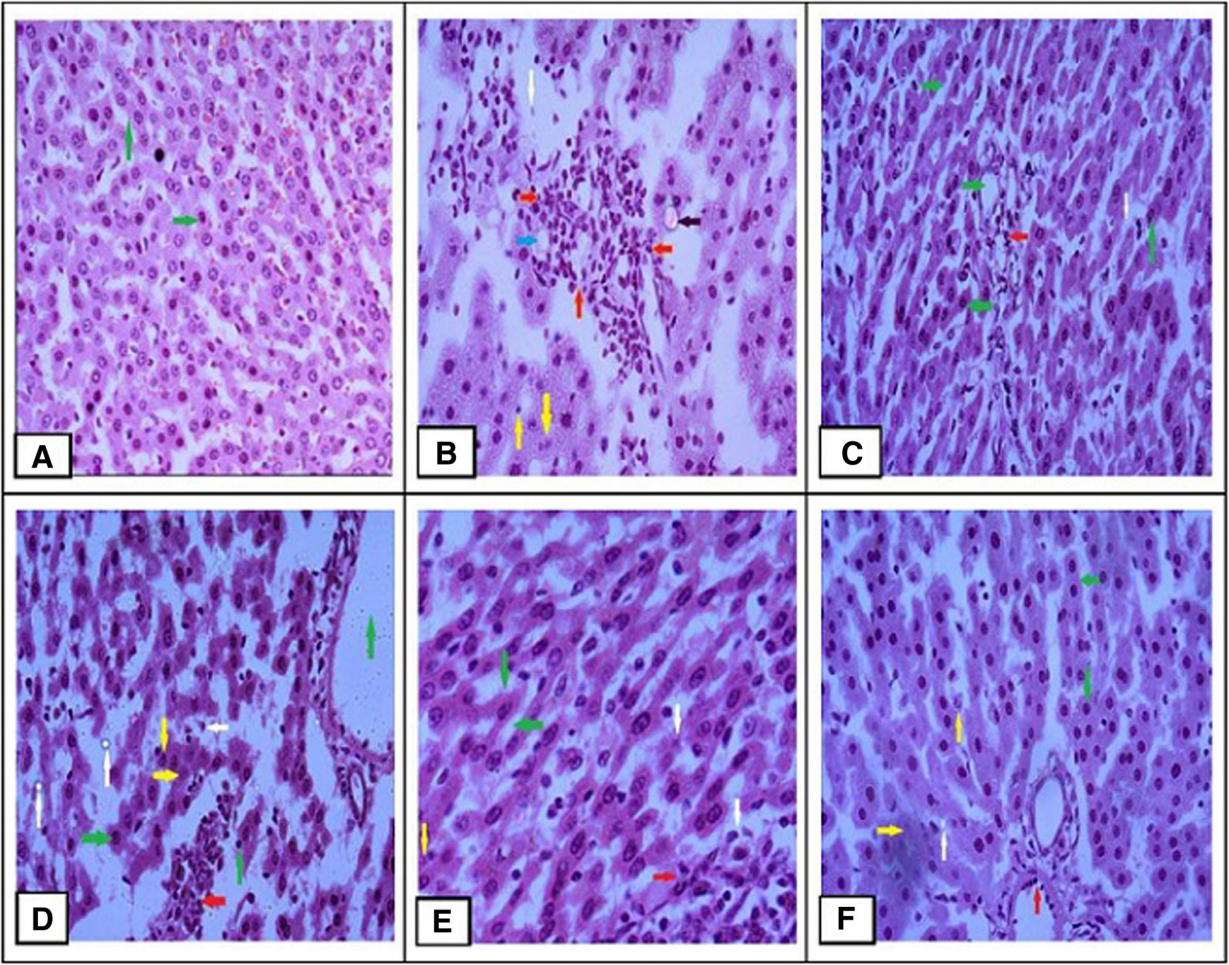
Histopathological observation of liver samples. **a** N-C group showed normal arrangement of hepatocytes and sinusoids (Green arrow) (H&E ×20); **b** N-Cd group showed infiltration of the portal tract with inflammatory cells (Red arrow), vacuolation of liver cell (Blue arrow), hydropic degeneration (Black arrow), nuclear pyknosis and karyorrhexis (Yellow arrow), and fibrosis (White arrow) (H&E ×40); **c** N-P group showed hepatocytes with regular and large spheroidal nucleus (Green arrow), normal sinusoids (Green arrow), mild inflammatory cells (Red arrow) and low fat deposition (White arrow) (H&E × 20); **d** N-PCd group showed some normal hepatocytes with central vein (Green arrow), less inflammation (Red arrow), fatty changes and fibrosis (White arrow), karyorrhexis and karyolysis (Yellow arrow) (H&E × 40); **e** DM-P group showed normal appearance of hepatocytes (Green arrow), mild inflammation (Red arrow), fibrosis (White arrow) and nuclear pyknosis (Yellow arrow) (H&E x 40); **f** DM-PCd group showed restoration of hepatocytes (Green arrow), karyorrhexis and karyolysis (Yellow arrow), reduced both inflammation (Red arrow) and fatty changes (White arrow) (H&E × 40)

**Fig. 2:**
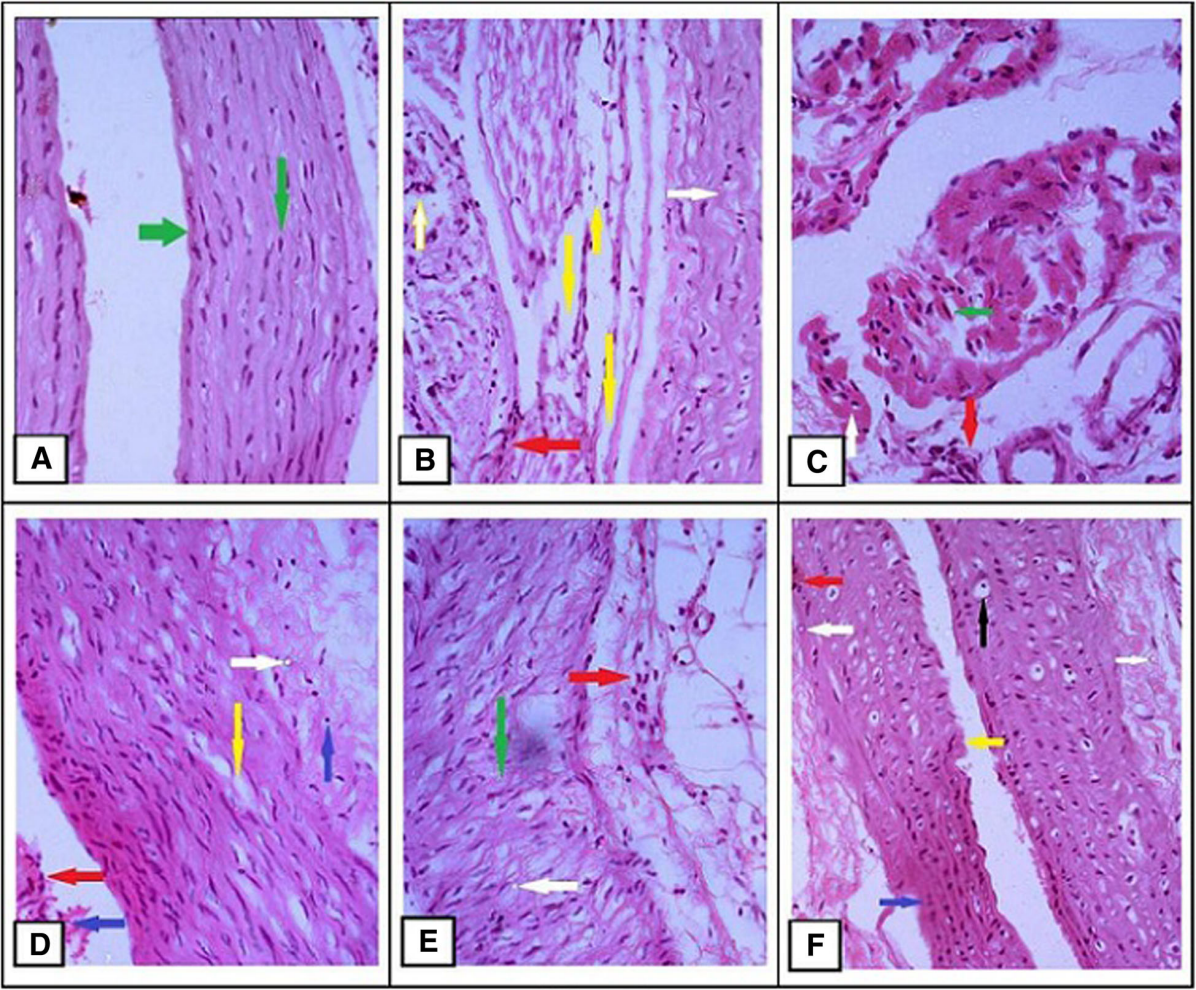
Histopathological observation of heart samples. Histopathological observation of heart in the study groups **a** N-C group showed normal myocardial fibers with membrane integrity (Green arrow) (H&E × 20); **b** N-Cd group showed inflammation (Red arrow), severe loss of myocardial fibers and hyalinization (Yellow arrow), and fatty changes (White arrow) (H&E × 20); **c** N-P group showed normal myocytes (Green arrow), mild inflammation (Red arrow) and low fat deposition (White arrow) (H&E × 40); **d** N-PCd group showed inflammation (Red arrow), less irregularity of myofibrils (Yellow arrow), fat deposition (White arrow), nuclear pyknosis and necrosis (Blue arrow) (H&E × 20); **e** DM-P group showed mild inflammation (Red arrow) including fatty changes (White arrow), and normal myocardial fibers (Green arrow) (H&E × 20); **f** DM-PCd group showed less inflammatory cells (Red arrow), vacuolization of cytoplasm (Black arrow), myocardial fibers loss (Yellow arrow), steatosis (White arrow) and less necrosis (Blue arrow) (H&E × 20)

The liver and heart histopathological investigations were showed in Figs. 1 and 2. Compared to the control group (Fig. 1 a) there were severe inflammatory cells, hydropic degeneration, vacuolation of liver cell, nuclear pyknosis, karyorrhexis, fatty changes, and fibrosis were found in the Cd treated groups (Fig. 1 b and d). No major abnormalities were found in the plant extract treated group (Fig. 1 c). Similarly, the protective effects of the plant were observed in the DM-P group (Fig. 1e), which exhibited mild nuclear pyknosis, less inflammation and fibrosis compared to the DM-PCd group (Fig. 1 f). The histopathology of arch of aorta showed normal architecture in control rats (Fig. 2 a). Various abnormalities including loss of myocardial fibers, hyalinization, vacuolization of cytoplasm, nuclear pyknosis, necrosis, and fatty changes were clearly demonstrated in the N-Cd and N-PCd groups (Fig. 2 b and d). On the other hand, normal myocytes with mild inflammation and low fat deposition were observed in the plant extract treated group (Fig. 2 c). There are no major abnormalities were found in the DM-P group (Fig. 2 e) compared to the DMP-Cd which demonstrated inflammatory cells, vacuolization of cytoplasm, myocardial fibers loss, steatosis and less necrosis (Fig. 2 f). Thus, the plant extracts might confer some sort of protective activities against cadmium-induced toxicity.

## Discussion

Type 2 diabetes is a heterogeneous disease that imposes a heavy burden on global health through microvascular and macrovascular complications [3, 4]. The metabolic outcomes of this disease deteriorate in the presence of cadmium [14, 20]. In this study, we initially identified antidiabetic and antidyslipidemic effects of *Enhydra fluctuans* extracts in normal and type 2 model rats. We further explored antidiabetic efficacy of the plant in Cd toxicity.

The present study revealed that the ethanolic extracts of *E. fluctuans* at a dose of 200 mg/kg significantly reduced fasting blood glucose level (*p* < 0.05) in normal (N-PCd) and diabetic (DM-P, DM-PCd) rat models (Table 1) and it was more pronounced in merged-diabetic group (*p* < 0.001) (data were not shown). The antidiabetic effects were further confirmed by comparing within and between the study groups (Table 2). In both ways, the plant showed significant antihyperglycemic activity and it was preserved even under Cd induced condition. Our results are in agreement with reported literature from traditional medicine practitioners that *E. fluctuans* exhibits antidiabetic activity [32, 46]. This effect is attributed to the various agents present in the plant that may enhance glucose uptake and prevent hyperglycemia through several mechanisms such as increased inhibitory effects against insulinase, reduced hepatic inflammation and improved insulin sensitivity [28, 31, 47]. As well as other mechanisms also involved such as inhibition of intestinal glucose absorption, enhanced peripheral glucose utilization, hepatic glycogen synthesis or decrease of glycogenolysis [30, 31, 47, 48]. In contrast, CdCl_2_ alone had no effect on glucose metabolism. This study is in consistent with the findings of reported literature that short time exposure of Cd seems to have no direct effects on glucose metabolism. Its effects may come from chronic exposure by pancreatic impairment and insulin resistance in multiple peripheral tissues [16]. Our study found direct effects on body weight reduction, liver and kidney impairment, which may be due to the higher dose of CdCl2 induction for consecutive 21 days. Previous studies have also shown that Cd treatment reduced body weight gain and increased liver dysfunction in a dose dependent manner [13, 49]. Impaired hepatic function induced by Cd prevents lowering of blood glucose and TG/HDL-C ratio leading to worsened metabolic regulation [16, 18]. This was clearly demonstrated from the association of serum glucose, GPT and TG/HDL-C ratio in N-PCd, N-P, DM-P and the merged group (Table 5). It was also evident from histological diagnosis of hepatocytes that show severe inflammatory cells, hydropic degeneration, vacuolation, nuclear pyknosis, karyorrhexis, and fibrosis in the Cd treated groups (Fig. 1). One possible mechanism of hepatotoxicity is the oxidation state in which Cd can replace zinc present in metallothionein, thereby inhibiting it from acting as a free radical scavenger within the cell [50]. The histology of the arch of the aorta showed that plant treatment protects from vascular degradation, vacuolization of cytoplasm, hyalinization, necrosis and fibrosis (Fig. 2). This similar protective effects of *Tinospora cordifolia* (TCME) on Cd-induced cardiotoxicity prevented the alteration of serum marker enzymes (creatine kinase and lactate dehydrogenase), antioxidants, and glycoproteins contents [51]. Therefore, *E fluctuans* may have potent agents that could directly ameliorate glucose metabolism and partially neutralize toxicities generated by Cadmium.

The plant extract did not show any direct effect on total blood lipids in the study. However, most of the plant treated groups showed a reduced ratio of TG/HDL-C (Table 3), indicating the potential improvement of insulin sensitivity in diabetes. While the increased TG/HDL-C represented central obesity, which is directly associated with insulin resistant by altering secretion of different adipocytokines namely adiponectin, leptin, resistin and visfatin [52]. In addition to insulin resistance, TG/HDL-C ratio could predict accurately the risk for CHD and CVD mortality [52, 53]. These results are in good correlation with earlier reports, which stated that high triglycerides with low HDL-C leads to diabetes onset [54]. However, our study was unable to inhibit individual lipid parameters [55] and failed to reduce TG/HDL-C ratio in DM-P group. Despite these limitations, our plant improved TG/HDL-C ratio even in subjects with normal lipid profile. Hence, the plant could ameliorate metabolic regulation at the early stage of the development of metabolic disorders.

Although cadmium concentration was not measured in the plant extract, it has not shown any adverse effects on body weight loss, liver and kidney impairment both in normal and diabetic models. The presence of ascorbic acid, phenolics and flavonoids in *Enhydra fluctuans* extracts stimulates redox parameters and inhibits lipid peroxidation, protein carbonylation, intrinsic and extrinsic apoptotic markers which were responsible for overall protective effect [47, 48]. Lack of diabetic controls and standard drug group was a limitation in our study.

## Conclusion

We present a plant with potential antihyperglycemic and partial antihyperlipidemic activities in both diabetes and Cd toxicity conditions. This plant is able to ameliorate the synergistic action of Cd and diabetes using various phyto-chemical compounds possess in the plant. Our findings provided scientific confirmation for the safe use of *E. fluctuans* by traditional healers in the treatment of diabetes. This study would enable additional efforts to move *E. fluctuans* closer to clinical relevance.

## Data Availability

The author has the availability of data and material in the selected repository.

## Abbreviations

ALT: Alanine amino transferase
Bw: Body weight
CdCl_2_: Cadmium
Chol: Cholesterol
DM: Diabetic Mellitus
DM-P: Diabetic Mellitus Plant treated
DM-PCd: Diabetic Mellitus Plant + Cadmium (CdCl_2_) treated
DMSO: Dimethyl sulfoxide
FSG: Fasting Serum Glucose
H&E: Haematoxylin and Eosin
HDL: High-Density Lipoprotein
N-C: Normal water control
N-Cd: Normal Cadmium (CdCl_2_)
N-P: Normal plant treated
N-PCd: Normal plant + Cadmium (CdCl_2_) treated
SGPT: Serum Glutamic Pyruvic Transaminase
STZ: Streptozotocin
TC: Total Cholesterol
TG: Triglycerides
